# Enzymatic Hydroesterification of Waste Oils: Combilipase-Assisted Ethyl Ester Synthesis via a Dual-Step Biocatalytic Process

**DOI:** 10.1021/acsomega.5c01442

**Published:** 2025-05-16

**Authors:** José Gadelha Lima Neto, Paulo Gonçalves de Sousa Júnior, Dayana Nascimento Dari, Francisco Izaias da Silva Aires, Kaiany Moreira dos Santos, Viviane de Castro Bizerra, Patrick da Silva Sousa, Francisco Simão Neto, Maria Alexsandra de Sousa Rios, Diego Lomonaco, Aluísio Marques da Fonseca, José Cleiton Sousa dos Santos

**Affiliations:** † Departamento de Engenharia Química, 28121 Universidade Federal do Ceará, Campus do Pici, Bloco 709, Fortaleza, Ceará CEP60455760, Brazil; ‡ Departamento de Química Analítica e Físico-Química, Universidade Federal do Ceará, Campus do Pici, Bloco 940, Fortaleza, Ceará CEP 60455760, Brazil; § Instituto de Engenharias e Desenvolvimento Sustentável, Universidade da Integração Internacional da Lusofonia Afro-Brasileira, 245069 Campus das Auroras, Redenção, Ceará CEP 62790970, Brazil; ∥ Departamento de Química Orgânica e Inorgânica, 308253 Universidade Federal do Ceará, Fortaleza, Ceará 60440-900, Brasil

## Abstract

Hydroesterification is a synthetic route involving hydrolysis followed by esterification. This study aimed to investigate the production of ethyl esters via enzymatic hydroesterification through theoretical and experimental approaches. Complete hydrolysis of residual frying oil was achieved using a 1:1 mass solution at 40 °C for 4 h with 0.4% Eversa Transform 2.0 lipase relative to the oil mass. A combination of Eversa Transform 2.0 and lipase B from *Candida antarctica* (CAL B) was used in the esterification step. A Taguchi statistical design evaluated the effects of enzyme combination (1:1, 1:2, 1:3), FFA/alcohol molar ratio (1:1, 1:8, 1:15), biocatalyst percentage (5%, 10%, 15%), and reaction time (2, 4, 6 h). Optimal conditions were identified as a 1:8 molar ratio (FFA/ethanol), 10% biocatalyst, and a 1:3 enzyme combination for 6 h, theoretically yielding 80.1 ± 0.02% conversion. Experimentally, a 71.4 ± 0.1% conversion was achieved, slightly lower due to biocatalyst susceptibility to interferences affecting catalytic activity. Viscosity and density analyses confirmed the potential of the produced ethyl esters for future applications. This combined theoretical and experimental study highlights the feasibility of enzymatic hydroesterification using waste frying oil and combilipases as a sustainable approach for biodiesel production.

## Graphical abstract

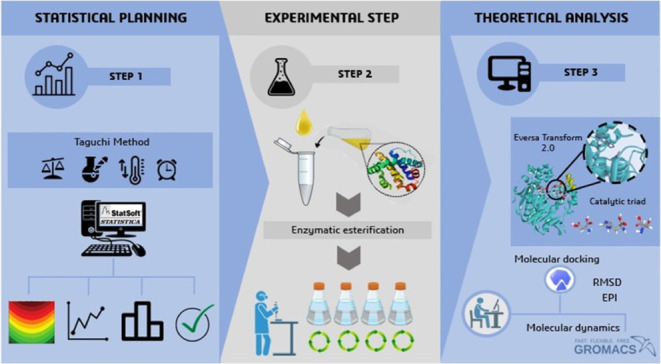

## 1 Introduction

Biodiesel is a prominent example of a renewable fuel, as it may replace diesel wholly or partially in internal combustion engines.^1–3^ Biodiesel can be produced from various sources, including vegetable oils, animal fats, and waste oils.^4–6^ These oil sources are composed of fatty acids and glycerol, forming glycerides. It is important to emphasize that the physiological characteristics of oilseed sources significantly influence the oil’s properties and, consequently, the physicochemical characteristics of the resulting biodiesel.^7–9^

Recent advances in biodiesel production have focused on optimizing heterogeneous practices, using unconventional raw materials, and applying more sustainable technologies.^10–12^ Biochar- and hydrochar-based developments show promise, enabling more efficient and proportionate responses to waste.^13–15^ In addition, the use of residual oils and lignocellulosic biomass as lipid sources has been improved, increasing the dependence on edible vegetable oils.^16–18^

In Brazil, it is estimated that approximately 3 to 4 billion liters of vegetable oil are generated annually for culinary use. However, only about 1% of this volume is correctly disposed of, and the rest is released into the environment with no appropriate treatment. Disposing residual oil from the frying process causes several environmental problems (Figure 1), including the contamination of water bodies and damage to their ecosystems.^19,20^

**Figure 1:**
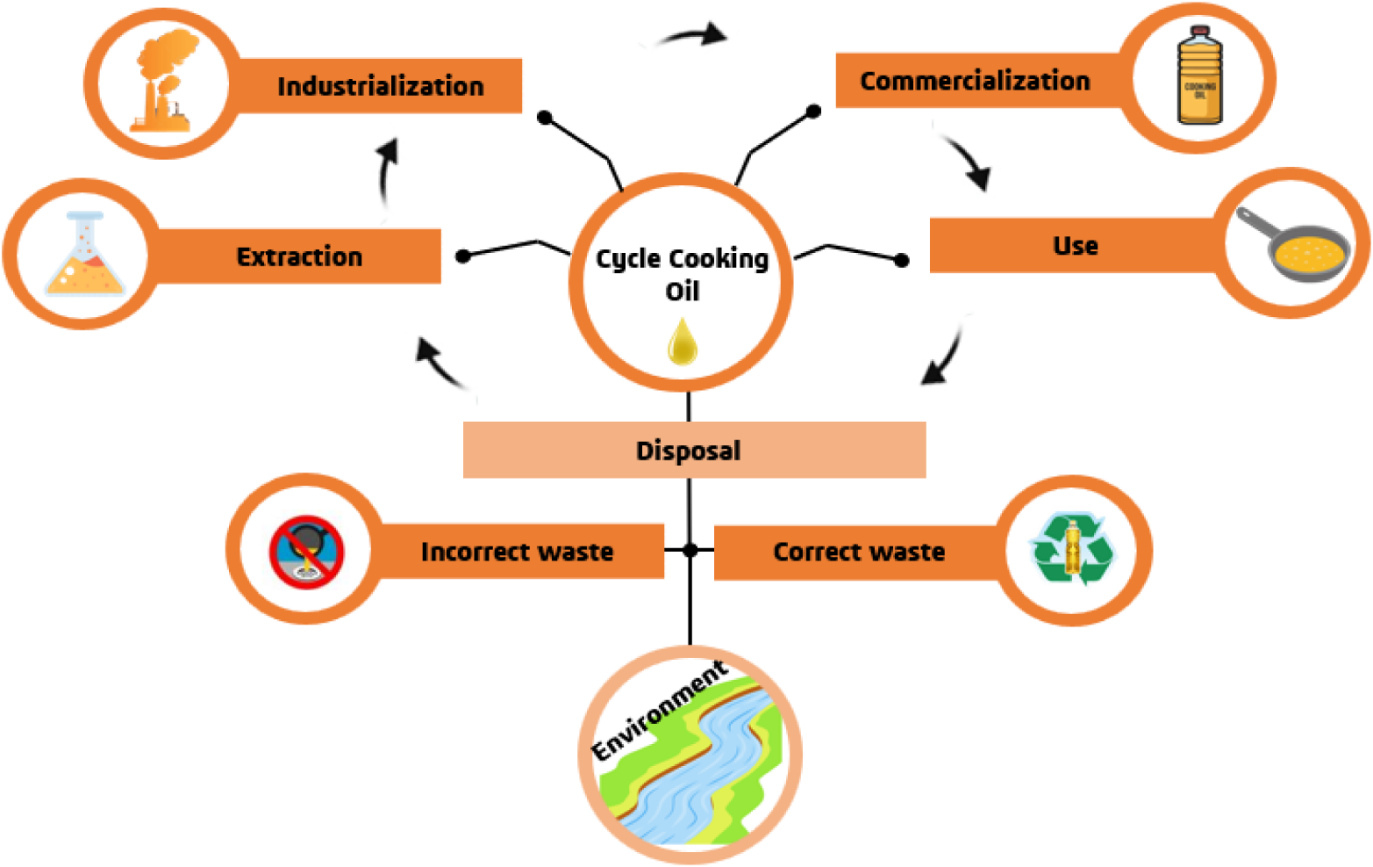
Representation of the cooking oil cycle, from preparation processes to waste disposal.

Although cooking oil is environmentally harmful, it can be an excellent byproduct for the production chain. Cooking oil can be collected in cafeterias and industrial or domestic kitchens. Burnt cooking oil can be given a proper destination through reuse and recycling, potentially generating biofuels.^21,22^ The effects of different sources of waste cooking oil, such as various types of vegetable oils or animal fats, on reaction efficiency, can be significant, depending on the chemical properties of the oils and fats used.^23,24^ These differences in fatty acid composition can influence the reactivity and efficiency of reactions, such as transesterification (in biodiesel production) or other chemical conversions.^25,26^

An alternative to solve the problems caused by inadequate cooking oil disposal would be to replace it with raw material.^27–29^ Aside from thermal cracking, producing ethyl esters can be performed in the presence of off-chemical catalysts (homogeneous and heterogeneous) or enzymatic catalysts, offering various raw material alternatives.^30–32^ Enzymatic catalysts, in particular, stand out as a more viable alternative among the different available types, as they generate less waste and have a lower environmental impact than chemical catalysts.^33–35^

Enzyme catalysts stand out as promising biocatalysts with broad potential. Biocatalysts can operate under mild temperature and pressure conditions without altering their activity.^36–38^ In addition, they exhibit high selectivity and specificity and are biocompatible and biodegradable.^39^ However, the large-scale implementation of biocatalysts in industry faces challenges because of their high cost and limited operational stability.^40^ Hence, given this scenario, various strategies are being implemented to broaden the use of biocatalysts in the industrial sector.^41^ Techniques such as using green solvents for enzymes and genetic manipulation in enzyme production are emerging as economically viable alternatives.^42,43^

Enzymatic catalysis presents notable advantages over conventional acid- and base-catalyzed transesterification methods, particularly in converting residual oils with elevated free fatty acid (FFA) content.^44^ Although alkaline transesterification exhibits high conversion efficiencies, its susceptibility to FFAs leads to soap formation, necessitating a pretreatment step.^45^ Conversely, acid-catalyzed transesterification is less influenced by FFAs but is limited by prolonged reaction times and the increased corrosivity of the reagents.^46^ Enzymatic catalysis circumvents the need for oil pretreatment and operates under milder temperature and pH conditions, thereby reducing energy consumption and minimizing the generation of undesirable byproducts.^47^

Using machine learning and computational chemistry to improve process efficiency and reduce costs,^48^ biodiesel production optimization has been explored. Machine learning models are applied to predict reaction yields, optimize operational parameters, and identify more efficient catalysts, reducing the need for empirical experimentation.^48–50^ In parallel, computational chemistry allows the simulation of molecular interactions, facilitating the rational design of more efficient catalysts and solvents.^51–53^ These approaches enable faster and more accurate development of new methods and improve biodiesel production’s economic and environmental viability.

Understanding enzymatic processes at the atomic and molecular levels facilitates the large-scale application of these materials.^54–56^ Advances in the development of high-capacity computers and software have promoted the growth and consolidation of computational chemistry and bioinformatics, which are critical to understanding processes at the atomic level.^57^ The exploration of combinations of lipases, each with substrate-specific specificities, represents an innovative strategy currently under investigation. This approach, known as ″combilipase,″ aims to enhance the efficiency of heterogeneous processes and is called “combilipase”.^58^

Esterification, a reversible equilibrium reaction, is influenced by temperature, reaction time, catalyst loading, and the molar ratio of ethanol to free fatty acid.^59^ Temperature increases the reaction rate but can shift the equilibrium at very high temperatures.^60^ Reaction time affects conversion, with longer times generally leading to higher yields.^61^ Catalyst loading accelerates the reaction but may increase costs.^62,63^ The ethanol-to-free fatty acid ratio favors ester formation, but excess ethanol may lead to undesirable byproducts.^64^ A detailed parametric study is needed to optimize the yield. Therefore, the present study aimed to assess the catalytic potential of lipase Eversa Transform 2.0 and CALB, a combi-lipase, in synthesizing ethyl esters from waste frying oil. Enzymatic hydroesterification produced ethyl esters because this method has a lower environmental impact than conventional routes.^65^

## 2 Materials and Methods

### 2.1 Materials

Lipase B from commercial *Candida antarctica* (CALB, 6.55 mg/mL) and commercial lipase Eversa Transform 2.0 (1200 mg/mL) from *Aspergillus oryzae* were jointly purchased both sourced from the company Sigma–Aldrich Brasil Ltd. (São Paulo, Brazil).^66^ All other chemical reagents were of analytical quality obtained from Synth (São Paulo, Brazil) and Vetec (São Paulo, Brazil) and were of analytical grade. The Statistica 10.0 software (Statsoft, Tulsa, USA) was used for the development and experimental design based on Taguchi methods. The residual frying oil used in this study was collected from a local Redenção, Ceará, Brazil market.

### 2.2 Hydroesterification

In this study, ethyl esters were obtained and produced from residual frying oil using an enzymatic hydroesterification process (Figure 2). Hydroesterification consists of a strategy that involves two distinct stages. In the first stage phase, free fatty acids are synthesized from refined residual frying oil through enzymatic hydrolysis, utilizing free lipase from Eversa Transform 2.0 as a biocatalyst (Figure 3). Figure 3 illustrates this process in detail, highlighting a classic mechanism of enzymatic hydrolysis mediated by covalent catalysis. This type of reaction is fundamental for several biotechnological processes, including lipid degradation, biodiesel production, and ester synthesis. In addition, the structure of the catalytic triad plays an essential role in the stabilization of reaction intermediates and the efficiency of catalysis.

**Figure 2:**
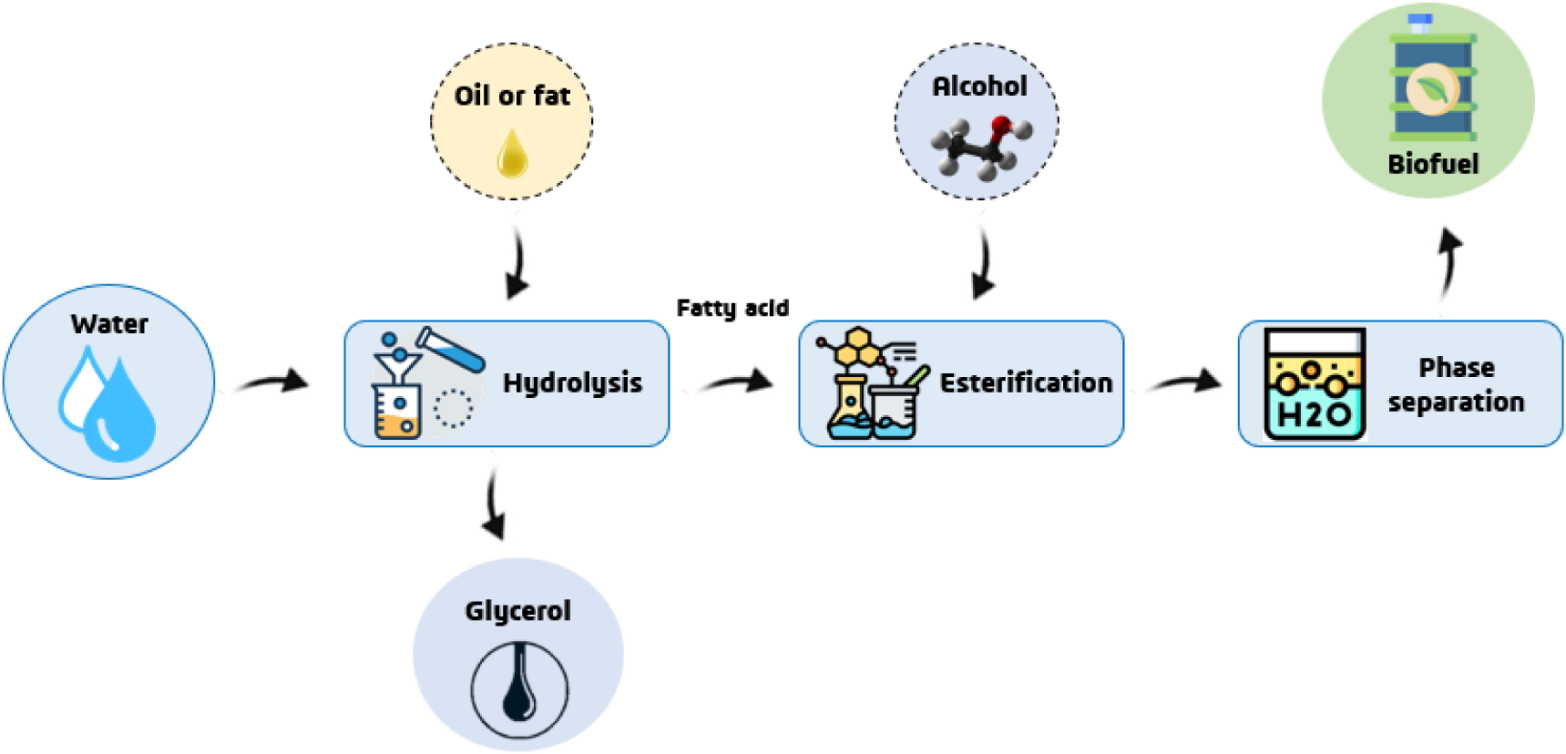
Diagrammatic representation of biodiesel synthesis by hydroesterification.

**Figure 3:**
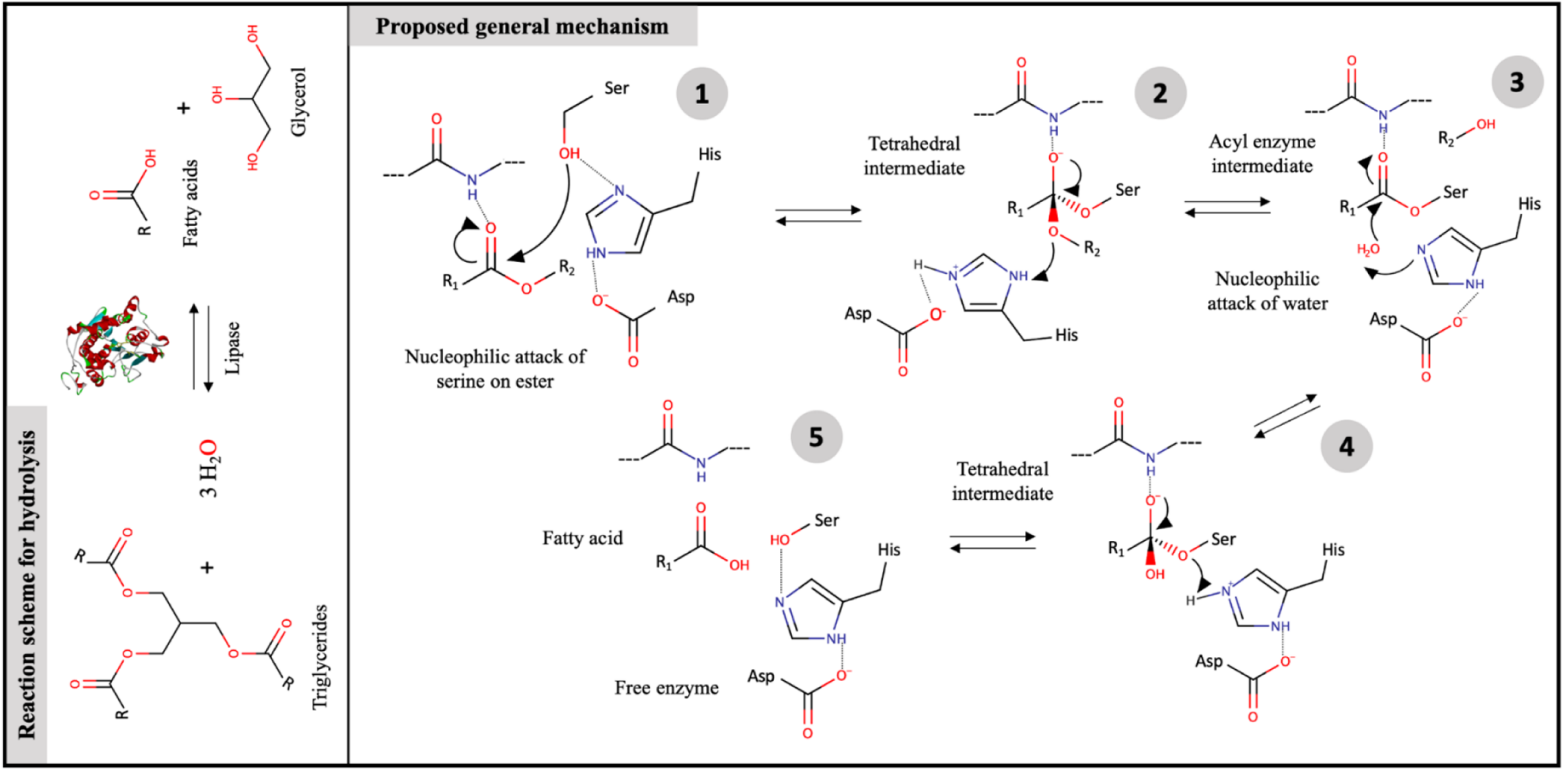
A general hydrolysis reaction scheme highlights the proposed mechanism, where the catalytic triad interacts with amino acid residues and substrate to form alcohol and acid.

The reaction system was adapted from the methodology proposed by^66^ with some modifications. Initially, the mixture containing oil and water in a 1:1 mass ratio was first heated until the system reached a temperature of 40 °C. Subsequently, 0.4% of the biocatalyst was added relative to the oil mass. The system was maintained at 40 °C for 4 h under constant stirring. The mixture was transferred to a separation funnel at the end of the process. Then, 100 mL of distilled water at 60 °C was added, creating a heterogeneous mixture that facilitated the separation of the aqueous phase from the free fatty acids (FFA) produced. After separation, the lower (aqueous) phase was discarded, and the FFAs were washed three times with distilled water three times.^67^ FFAs were then transferred into a beaker and heated to 80 °C for 10 min. After this period, the FFA was filtered using a funnel with filter paper containing anhydrous sodium sulfate (99%). This drying agent had been previously dried in an oven (SolidSteel-SSA, MG, Brazil) at 100 °C for 4 h.

The method for determining the initial acidity of FFA involved removing 2 g aliquots from the reaction supernatant and diluting them in 25 mL of an ethyl ether-ethanol solution (2:1 ratio). Then, three drops of phenolphthalein were added, and followed by filtration with a sodium hydroxide solution was performed (0.1M). To determine the initial and final acidity number (AI) of the FFA (after the hydrolysis step) of the FFA, we used an adapted version of the manuscript. The equation reported in the literature (eq 1) used^68,69^

1 AI(mgNaOH/g)=MMNaOH×MNaOH×f×((VNaOH)/m)

Where *MM*NaOH (g/mol) is the molar mass of NaOH; *M*NaOH (mol/L) is the molarity of the NaOH solution; *f* is the correction factor determined by NaOH standardization; *V*NaOH (mL) is the volume of NaOH used in the titration; and *m* (g) is the mass of the sample studied.

In the second stage (Figure 4), direct esterification of FFA derived from frying oil was conducted with ethanol, utilizing a combilipase composed of lipase Eversa Transform 2.0 and CALB in its free form as the biocatalyst. The reactions for producing ethyl esters of fatty acids were performed in 2 mL Eppendorf tubes with lids containing the biocatalyst and the substrate (i.e., FFA and ethanol). These tubes were placed on a rotary shaker (TE Incubator – 4200) with digital temperature and rotation control, set at 40 °C and 190 rpm. In this study, the variable parameters included enzyme combination ratios (1:1, 1:2, and 1:3), the molar ratio between FFA and ethanol (1:1, 1:8, and 1:15), the percentage of biocatalyst (5%, 10%, and 15%), and the reaction time (2, 4, and 6 h) according to the hours based on static design planning reported in previous studies.^70,71^

**Figure 4:**
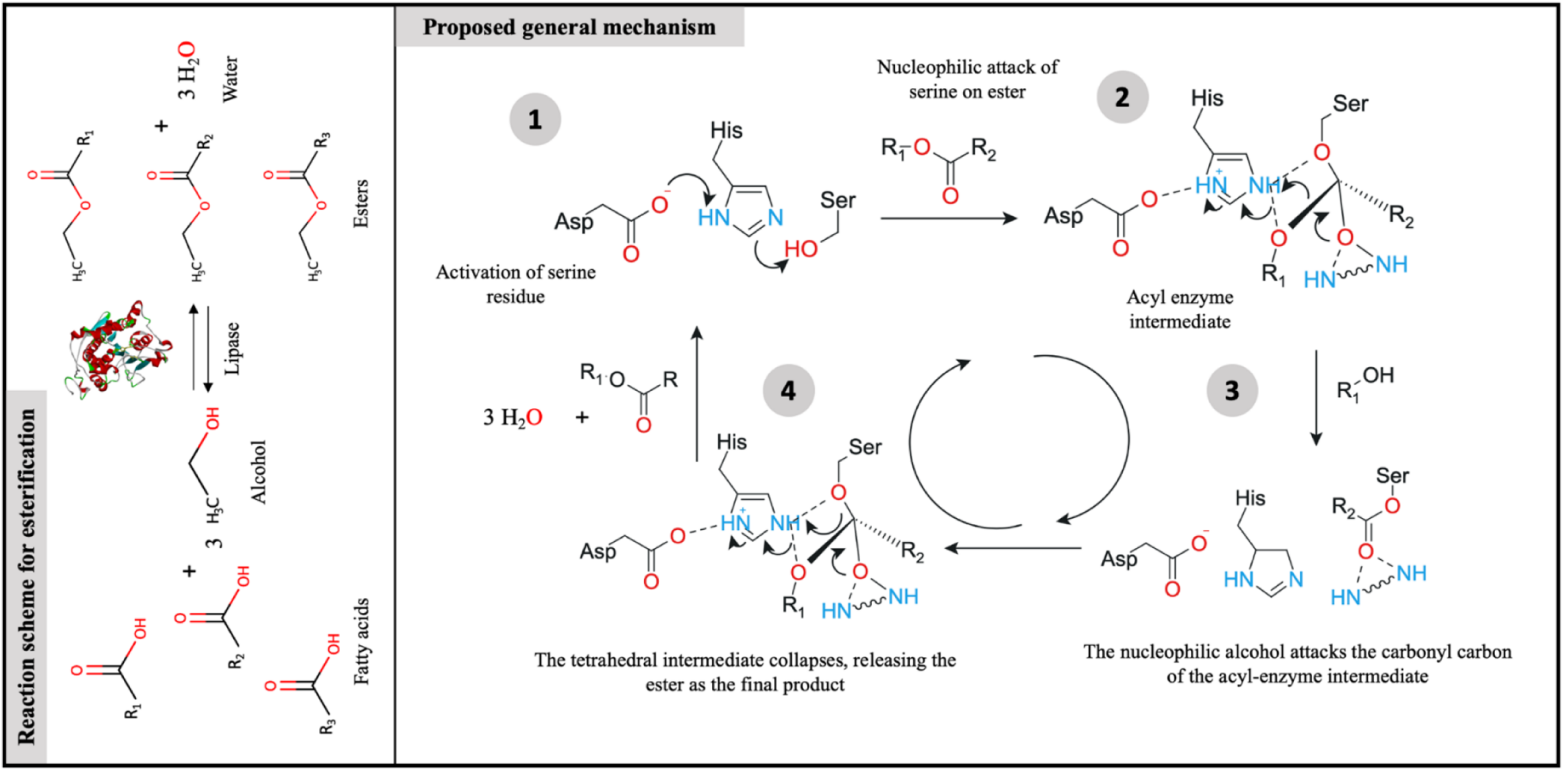
A general esterification reaction scheme highlights the proposed mechanism, where the catalytic triad interacts with amino acid residues and substrate to form an ester and water.

Equation 2 was used to calculate the conversion of free fatty acids to esters, considering the initial acidity of the sample (AIB) and the sample after the reaction (IAF).

2 $$\mathrm{AI}\left(\mathrm{mgNaOH}/g\right)=\frac{\mathrm{AIB}-\mathrm{AIF}}{\mathrm{AIB}}\times 100$$

The main advantages of this method are its simplicity and the production of biofuels with properties similar to those of diesel. However, significant challenges can arise when applying this strategy due to the need for high-purity reagents and pretreatment requirements.^72^

### 2.3 Physicochemical Analysis of Waste Cooking Oil

#### 2.3.1 Kinematic Viscosity

The biodiesel’s kinematic viscosity at 40 °C was measured according to ASTM D-7042. The analysis was conducted using an Anton Paar SVM 3000-Stabinger digital viscodensometer (São Paulo, Brazil) for analysis.^73^

#### 2.3.2 Density

The density of the oils at 20 °C was determined using the ASTM D-7042 standard. The analysis was performed with an Anton Paar SVM 3000-Stabinger digital viscodensometer (Graz, Austria), which was used for analysis.^74^

### 2.4 Characterization

#### 2.4.1 Analysis by Gas Chromatography (GC/MS)

GC/MS analysis was conducted according to the methodology described in standard EN 14.103,^75^ with modifications to determine the ester content of the residual cooking oil. Approximately 50 mg of the produced biodiesel obtained sample was added to a 2 mL vial containing 1 mL of methyl nonadecanoate solution (10 mg/mL). A 1 μL aliquot of this mixture was injected into a SHIMADZU QP-2010 ULTRA gas chromatograph–mass spectrometer (Kyoto, Japan), equipped with a capillary column (5%-phenyl)-methylpolysiloxane (DB-5) (30 m × 0 m, 25 mm ID × 0.25 μm film thickness), using helium as the carrier gas in splitless mode.^76^

#### 2.4.2 Nuclear Magnetic Resonance (NMR)

One-dimensional nuclear magnetic resonance spectroscopy of hydrogen (^1^H NMR) and carbon (^13^C NMR) was used to analyze the reaction intermediates and the final product. SpectraThe spectra were recorded using a Bruker Avance DRX500 spectrometer, model Advance DRX-500, at the Northeastern Center for the Application and Use of Nuclear Magnetic Resonance, located at the Federal University of Ceará (CENAUREMN-UFC). The experiment was conducted at a hydrogen frequency of 500 MHz to determine the chemical shifts and multiplicities of the isotopes in the carbon chains of the formed esters. Each sample was dissolved in deuterated chloroform (CDCl_3_) and analyzed in 5 mm tubes.

### 2.5 Taguchi Method

This research aimed to optimize the production of ethyl esters from waste cooking oil by using combilipases). The Taguchi design with a standard orthogonal matrix L9 (where L9 represents the Latin square and the number of experiments, respectively) was adopted to analyze four distinct factors at three levels to define. This approach aimed to determine the optimal reaction conditions for the efficient immobilization of these enzymes.^68^ Table 1 presents the corresponding levels of the four independent factors (i.e.,time, molar ratio, enzyme combination, and biocatalyst concentration.^77^

**Table 1:** Experimental Design: Three Coded Levels of Four Independent Parameters

	Time (h)	Combination (U/U)	Molar ratio (GL/AA)	Biocatalyst (% m/m)
**Level 1** **(L1)**	2	1:1	1:1	5
**Level 2 (L2)**	4	1:2	1:8	10
**Level 3 (L3)**	6	1:3	1:15	15

Statistica software was used for the experimental design and statistical analysis. Table 2 shows the experimental design.^78^

**Table 2:** Experimental Design of Taguchi L9 Planning Method

Exp	Time (h)	Combination (U/U)	Molar Ratio	Biocatalyst (% m/m)
**1**	2	5	01:01	01:01
**2**	4	10	01:01	01:02
**3**	6	15	01:01	01:03
**4**	6	5	01:08	01:02
**5**	2	10	01:08	01:03
**6**	4	15	01:08	01:01
**7**	4	5	01:15	01:03
**8**	6	10	01:15	01:01
**9**	2	15	01:15	01:02

The “higher is better” function was used to calculate the S/N ratio values corresponding to the biocatalyst activity values since the study’s objective was to maximize the response variable (biocatalyst activity). Calculate the signal-to-noise ratios for each experiment (eq 3):

3 $$\frac{S}{N}=-10\log\left(\frac{1}{n}\overset{n}{\underset{j=1}{\sum }}\frac{1}{y_{i}^{2}}\right)\times 100$$

In this context, *y* represents the response variables, *i* represents the number of replications, and *n* represents the number of experiments used for the factor-level combination of each design combination. We could use eq 4 to determine the expected S/N (signal-to-noise) ratio for the ideal conditions to achieve the optimal biocatalytic activity.

4 $$S/N_{\mathrm{prediction}}=S/N\overset{n}{\underset{j=1}{\sum }}\left(\frac{S}{N_{j}}-\frac{S}{N}\right)$$

S/N prediction is the arithmetic mean of all S/N ratios, S/N*_j_* is the S/N ratio at the ideal point for each factor, and *n* is the number of factors significantly affecting the process.^79^

### 2.6 *In Silico* Study

#### 2.6.1 Preparation of Binders and Proteins

The molecules of lipid composition of residual oil with combilipases CALB and Eversa 2.0 were generated by Chem3D software (Ahmadi et al. 2005)^79^ in auto-optimization settings, the MMFF94sMMFF94S force field was applied^80^ to create bioactive conformations by minimizing randomly generated conformers, with Steepest Descente Algorithm,^81^ Stepper Update 4,^82^ by using AVOGADRO software.^83^ The file containing the ligand file was converted to the appropriate corresponding format (.pdbqt) by adding ionization and tautomeric states at pH 7.4 by using 3.0.0 OpenBabel version 3.0.0 software.^84^

The receptor under study was the structure of CALB, obtained from the Protein Data Bank repository under code ID CALB (1TCA),^85^ and Eversa 2.0. Some procedures from literature,^86,87^ were modeled, whose crystalline structure was obtained by complex X-ray diffraction. To enable molecular docking, interfering residues, water molecules, and the synthetic inhibitor were removed. Polar hydrogens were added separately to both the ligands and to the protein. This technique searches within potential virtual ligand databases for a given protein target. The software used for this process was Autodock Tools software.^88^

#### 2.6.2 Molecular Docking

Molecular docking was performed using AutoDock Vina.^89^ Lamarkian Genetic was performed using 3-way multithreading. For the docking of the combilipases CALB lipase (1TCA) and Eversa 2.0 main protease, the following complex parameters were used: several grid points in *xyz* (30, 30, 30), spacing (0.642), grid center in *xyz* (−34.282188, 24.937188, 73.691625). Other parameters were set to default. Input ligands with polar hydrogens were used in .pdbqt format. Between 10 and 40 molecular docking run executions were performed, and several simulations were repeated in the same region of the biological receptor. Thus, to validate the simulation’s performance and quantify the quality of the dockings, the RMSD (root-mean-square deviation (rmsd) scoring criterion was used, which suggests that it was adopted. According to this criterion, a successful docking exhibits an rmsd value of ≤2.0 Å.^90^ Discovery Studio software^91^ and PyMOL^92^ were used to visualize the simulation data and to highlight the main receptor–ligand interactions.

### 2.7 Molecular Dynamic

Molecular dynamics (MD) simulations were performed using the NAMD program.^93^ Best conformations obtained in molecular coupling were solved in water in the TIP3P model,^94^ and in the CHARMM36 force field, ions were added to neutralize the total system charge. The system then underwent energy minimization using the Steepest Descent method. Subsequently, it was subjected to *NVT* (constant number of particles, volume, and temperature) and *NPT* scales (constant number of particles, pressure, and temperature) equilibrations under conditions described by Langevin dynamics.^95^ Simulations of the system fabrication production simulations were performed for 100 ns. The quality of the structures obtained from the molecular dynamics (MD) simulations was evaluated with NAMD using the following parameters with NAMD:

- Potential energy (kcal/mol);^96^
- Protein–ligand interaction energy (kcal/mol);
- The rmsd of the atomic positions of proteins, binders, and the distances between them (rmsd, Å), and the root-mean-square deviation of the nuclear positions of proteins, ligands, and the distances between them (rmsd, Å);
- Hydrogen bonds were evaluated with visual molecular dynamics (VMD);^97^
- The mean quadratic fluctuation of the minimum distances between proteins and ligands was observed in MD (RMSF, Å).^98^ The graphs were generated using the Qtrace software.^99^

## 3 Results and Discussion

### 3.1 Enzymatic Hydrolysis

The proposed enzymatic hydrolysis was successfully conducted, increasing the oil’s acid value from 6.57 mgNaOH/g to 82.86 mgNaOH/g. This increase in acidity is consistent with expectations, considering that refined residual frying oil was used during the hydrolysis process.^100^ The complete hydrolysis of the refined and residual oil was achieved in 3 h using lipase from *Candida rugosa* soluble as a biocatalyst. Since biocatalysts are environmentally friendly, enzymatic hydrolysis presents a viable alternative for this process.^21^

### 3.2 Esters Characterization

#### 3.2.1 NMR

In the ^1^H NMR spectrum (500 MHz, CDCl_3_) of the reaction medium, it is possible to observe the signals for the esters formed. Although the spectrum of the reaction mixture shows several overlapping typical peaks, the most significant peaks can be identified to determine the presence of these products in the reaction medium. Figure 5 displays some significant chemical shifts in ethyl oleate.^101^ Expansions for the hydrogens highlighted in the ethyl oleate structure were inserted in Figure 5 to identify and assign the signals to their respective chemical shifts and multiplicity values. Signal A has a chemical change close to δ 5.34 and was assigned to the hydrogens on the sp^2^ carbon. Due to its characteristic splitting for alkenes, this chemical profile leads to a signal with multiple characteristics. The B signal at δ 4.13 is a quadruplet, corresponding to the chemical multiplicity and shift attributed to the hydrogen-bonded directly bonded to the oxygen of the ester group. The peaks in region C have been attributed to the more diluted esters in the reaction medium, as indicated by their low intensity in the spectrum.^21^ The triplet observed in region D with a chemical shift of δ 2.77 is characteristic of methylene hydrogens in the α-carbonyl positions of esters. The signals observed in areas E and F are related to the methylene and methyl groups common to the esters present in the reaction medium. Finally, the G signal in G at δ 0.87 was attributed to the methyl group furthest from the functional group since it exhibited better shielding, a minor chemical shift, and corresponding diversity.^102^

**Figure 5:**
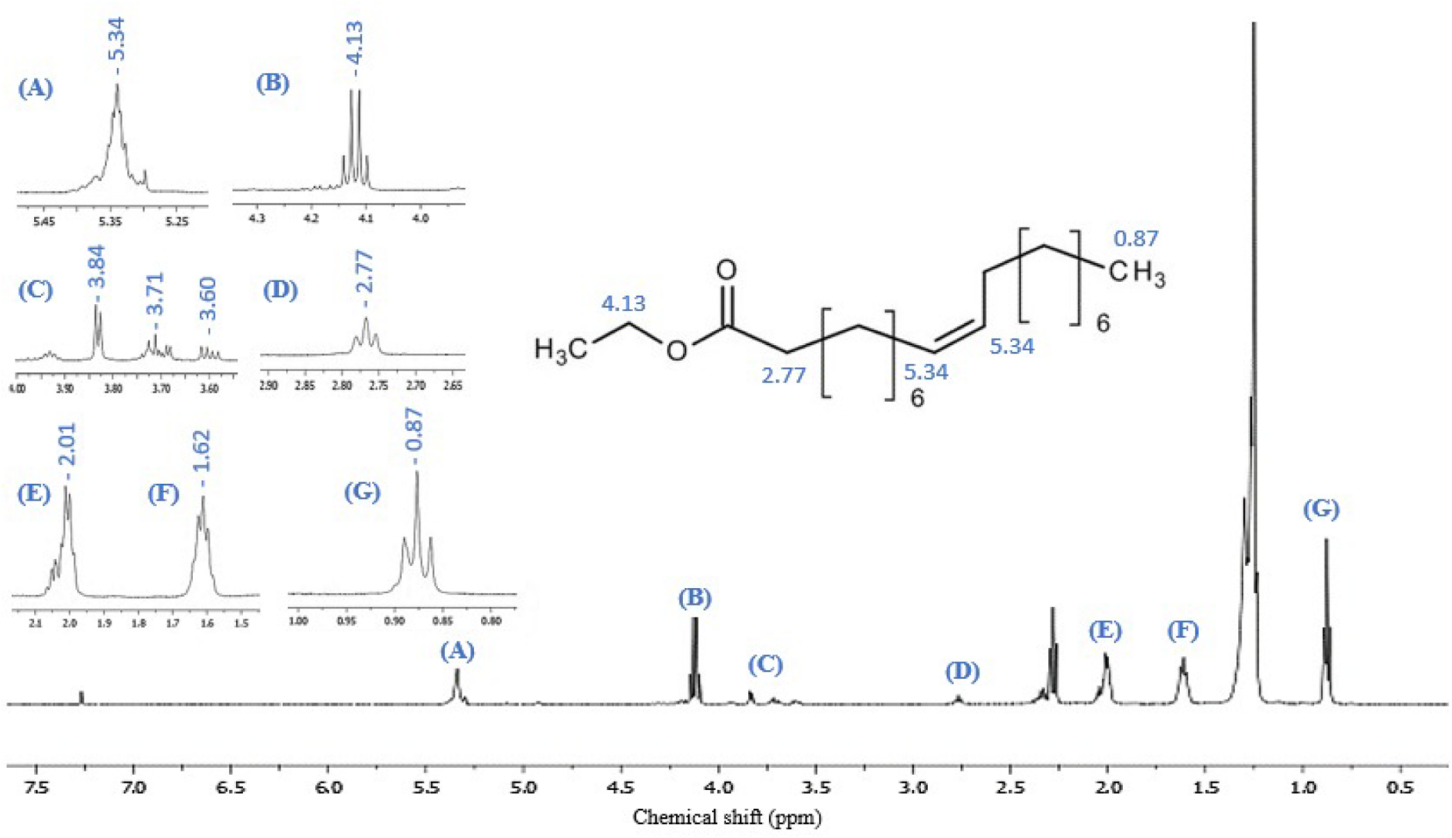
Hydrogen nuclear magnetic resonance (^1^H NMR) spectrum for the sample of the ester mixture with ethyl oleate expansions as the major product.

### 3.3 Taguchi Planning—Optimization of Ethyl Ester Production

At this stage of the work, the Taguchi design method with an L9 orthogonal matrix was used. This approach allows for analyzing parameter interactions and their effects onprocess.^103^ Table 3 presents the results obtained with the proposed reaction parameters. It is important to note that all experiments were conducted in triplicate, and the results were within the expected margin of error. Based on the data in Table 3, it was observed that test 5 yielded the best conversion and signal-to-noise ratio (S/N) results, with values of 80.0 ± 0.01 and 37.17, respectively. In this test, a 10% biocatalyst concentration was used, with a molar ratio of 1:8 (FFA/ethanol), over 2 h of reaction with an enzyme combination ratio of 01:03. The combilipase ratio of 01:03 significantly influenced the process. Among the parameters, the molar ratio had the least impact on the conversion values of ethyl esters. While its importance should not be excluded, some entirely dismissed, other factors proved to be more critical. Excessive use of alcohol can lead to enzyme denaturation or inactivation of the enzyme, thereby reducing the yield, resulting in reduced yields.^104^

**Table 3:** Experimental Design of the Taguchi L9 Planning

Reaction	Time (h)	Biocatalyst (% w/w)	Molar ratio (GL/AA)	Combination (U/U)	Conversion (%)	S/N
1	2	5	01:01	01:01	59.20 ± 0.97	36.86
2	4	10	01:01	01:02	76.40 ± 0.01	35.39
3	6	15	01:01	01:03	79.90 ± 0.07	37.15
4	6	5	01:08	01:02	62.50 ± 0.08	36.92
5	2	10	01:08	01:03	80.0 ± 0.01	37.17
6	4	15	01:08	01:01	77,80 ± 0,06	37.22
7	4	5	01:15	01:03	59.90 ± 0.03	39.52
8	6	10	01:15	01:01	74.90 ± 0.07	37.54
9	2	15	01:15	01:02	71.30 ± 0.18	36.86

### 3.4 S/N Ratio Analysis

Taguchi’s design uses planning to utilize S/N ratios to determine and assess the impact of various parameters on the process. In the present study, the ″bigger is better″ function was applied to calculate the S/N ratios, as the current design aim was to achieve higher conversion rates.^105^ Figure 6 illustrates the impact of each parameter at their respective levels. Table 3 provides the values depicted in Figure 6 and the Delta values, which are calculated by comparing the highest and lowest S/N ratios for each factor. By analyzing the Delta values, the influence of each parameter level on the process, and thereby identifying the parameters that can be assessed, enabling the identification of the most significant impact factors.^106^

**Figure 6:**
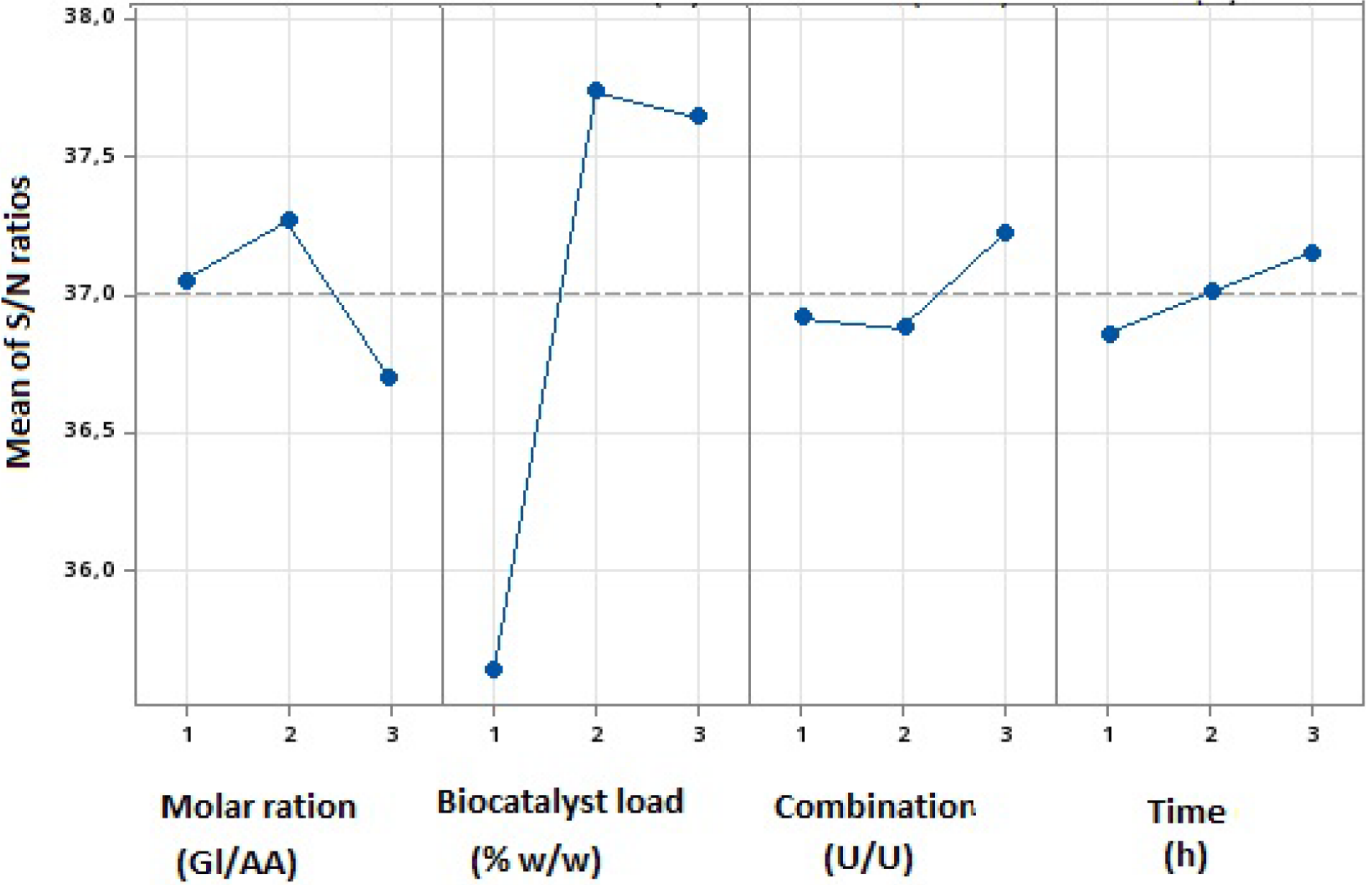
This means the average plot of parameter variation for each parameter studied.

The amount of biocatalyst was the most influential variable in the process, as indicated by the highest delta value of 2.10. The data in Table 4 confirms the significant impact of biocatalysts. The percentage increase in biocatalyst concentration in the reaction medium from level 1 (5%) to level 3 (15%) resulted in a substantial rise in the S/N ratio, response, and conversion efficiency. This improvement can be attributed to the more significant number of catalytic sites in the reaction medium. This availability is related to more coincidences resulting in a higher frequency of successful reactions.^107^

**Table 4:** Ranking of Variables Based on the Response to the Averages of the S/N Ratios

Factors/levels	Time (h)	Combination (U/U)	Molar ratio (AG/AA)	Biocatalyst (% w/w)
1	36.86	36.92	37.05	35.64
2	37.01	36.88	37.27	37.74
3	37.15	37.22	36.70	37.64
Delta	0.30	0.34	0.57	2.10
Ranking	4	3	2	1

### 3.5 Statistical Analysis

The conversion data collected through experimental design planning were evaluated using analysis of variance (ANOVA). According to the literature, the *p*-value determines the significance of each factor for the studied process. Table 5 shows the results obtained in the ANOVA.^108,109^

**Table 5:** Analysis of Variance (ANOVA) Results for Parameters Affecting Ethyl Ester Production

Factor	SS	DF	MS	*F* value	*p* value
**Time**	38.70	1	7.70	0.17	0.698
**Combination**	299.30	1	10.42	0.23	0.653
**Molar ratio**	67.62	1	14.727	0.33	0.595
**Biocatalyst**	853.63	1	374.460	8.44	0.044
**Waste**	177.381	4	44.34		
**Total**	**584.676**	8	-	-	-

To confirm a factor’s significance at a 95% confidence interval, the p-value must be under 0.05. Table 4 shows that only the percentage of biocatalysts was significant, with a *p*-value of 0.044. After analyzing the data, the optimal conditions for producing ethyl esters were determined based on levels (1, 2, and 3) of each factor (time, lipases, molar ratio [FFA/ethanol], and biocatalyst percentage) proposed in the L9 orthogonal Taguchi planning. Table 6 lists the optimal quantities for each factor level.

**Table 6:** Optimal Conditions for Producing Ethyl Esters from Waste Cooking Oil Are Statistically Defined

**Parameter**	**Molar ratio** **(GL/AA)**	**Biocatalyst** **(% w/w)**	**Combination** **(U/U)**	**Time** (h)
**Level**	2	2	3	3
**Value**	1:8	10	01:03	6

Theoretically, the optimum point would represent optimal conditions expected to yield a conversion rate of 88.26%. However, when these conditions were applied experimentally, the conversion achieved was 80.1% ± 0.02%. Despite this discrepancy, the methodology effectively approaches the optimization goal.

### 3.6 *In Silico* Study

#### 3.6.1 Molecular Docking

Oleic acid and CALB/Eversa lipases were used for molecular docking as per the literature and with some adaptations (Fujikoshi, 1993;^108^ Yves Nunes Holanda;^109^), oleic acid and *CALB/Eversa* lipases.

Affinities: The docking analysis performed using AutoDock Vina allowed for determining the affinity and RMSD energies of the ligands listed using the AutoDock Vina docking molecule (as presented in Table 7).

**Table 7:** Molecular Docking Result with Its Dock Scores

Compounds	PubChem	Energy (kcal/mol)	rmsd
dodecanoic acid	CID3893	–4.7	2.000
tetradecanoic acid	CID11005	–4.7	1.714
hexadecenoic acid	CID985	–5.7	2.000
oleic acid	CID445639	–5.8	2.000
octadecanoic acid	CID5281	–5.6	2.000

Although all ligands are allocated in the same region of the protein’s active site, which typically consists of the catalytic triad (Ser-His-Asp), the simulation for CALB lipase suggests a composition of Asp187-His224-Ser105 residues^110^ and the Eversa 2.0 Ser 153, His 268, and Asp 206.^111^

NACs (Near Attack Conformations) are conformations compatible with the catalytic site’s attack on the electrophilic carbon of the acyl group.^112^ In a typical NAC, the distance between the oxygen from the Ser 105 (CALB) or Ser 153 (Eversa) residue and the carbonyl carbon is usually observed to be close to 3 Å. The angle between these atoms and the carbonyl oxygen molecule forms an angle of about 60°, approximately 60°, with a maximum of 90°.^113^

In the present computational study, for the CALB lipase region, one direct interaction was observed in all 10 poses of each substrate that configured an NAC. Nevertheless, the best interactions, which are considered closest to the enzyme’s active site, are highlighted. For the complex in the Eversa 2.0 region, four interactions were observed that configured NAC.

Initially, an approximation of tetradecanoic acid and the Eversa 2.0 region was observed in molecular docking. However, NAC was not configured close to the catalytic triad. Figure 7 shows a hydrogen bond with Tyr 29 (2.17 Å) because the approximation was between the carbonyl of the acid and the residue. There were also hydrophobic interactions of the alkyl type with the residues Tyr 92, Leu 283, Phe 265, Ile 94, and finally with the residue Leu 262.

**Figure 7:**
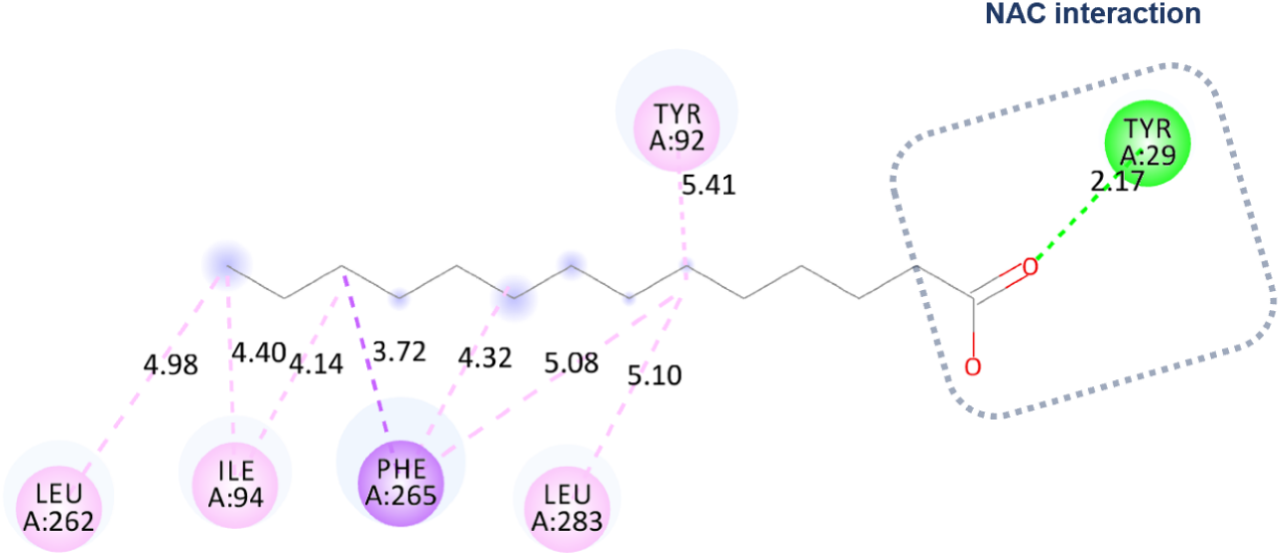
Molecular docking: approximation of tetradecanoic acid and the Eversa 2.0 region without configuring the approximation with the catalytic triad.

Molecular docking approximated the Eversa 2.0 region for the oleic acid ligand, with an NAC very close to the catalytic triad. Nevertheless, the main pose did not directly interact with the residues (Figure 8). There is a hydrogen bond with Tyr 29 (2.13Å) because the approximation was between the carbonyl of the acid and the residue. There were also hydrophobic interactions of the alkyl type with the residues Tyr 92, Ile 94, Phe 265, Leu 262, Val 269, and finally with the residue Leu 283.

**Figure 8:**
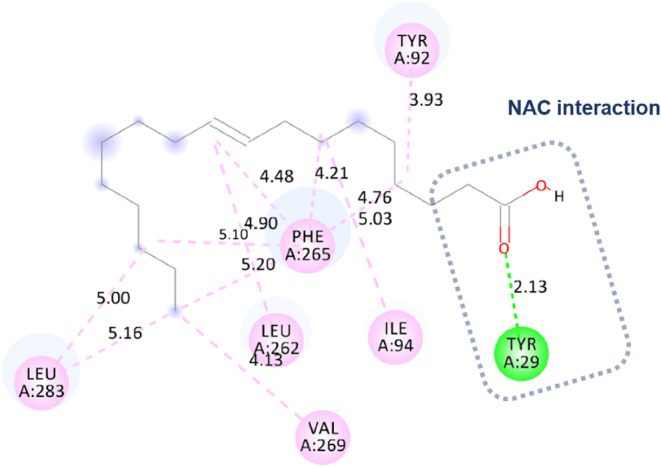
Molecular docking: approximation of oleic acid and the Eversa 2.0 region, with NAC very close to the catalytic triad.

In this simulation, for the region of the Eversa 2.0 lipase with octadecanoic acid, the results showed an approximation of the catalytic site of the lipase within the region area where the hydrolysis would be performed. Consequently, the 10 coupling position docking poses were classified as strong NACs because the distance between Ser-O (153) and the carbonyl-C of the substrate was less than 3 Å, with an angle less than 60° (Figure 9).^113^ In addition, another hydrogen bond was observed with the triad residue, His 268 (2.73 Å). The other interactions observed were hydrophobic and alkyl type with Tyr 92, Leu 262, Phe 265, Ile 94, Leu 283, and Leu 285.

**Figure 9:**
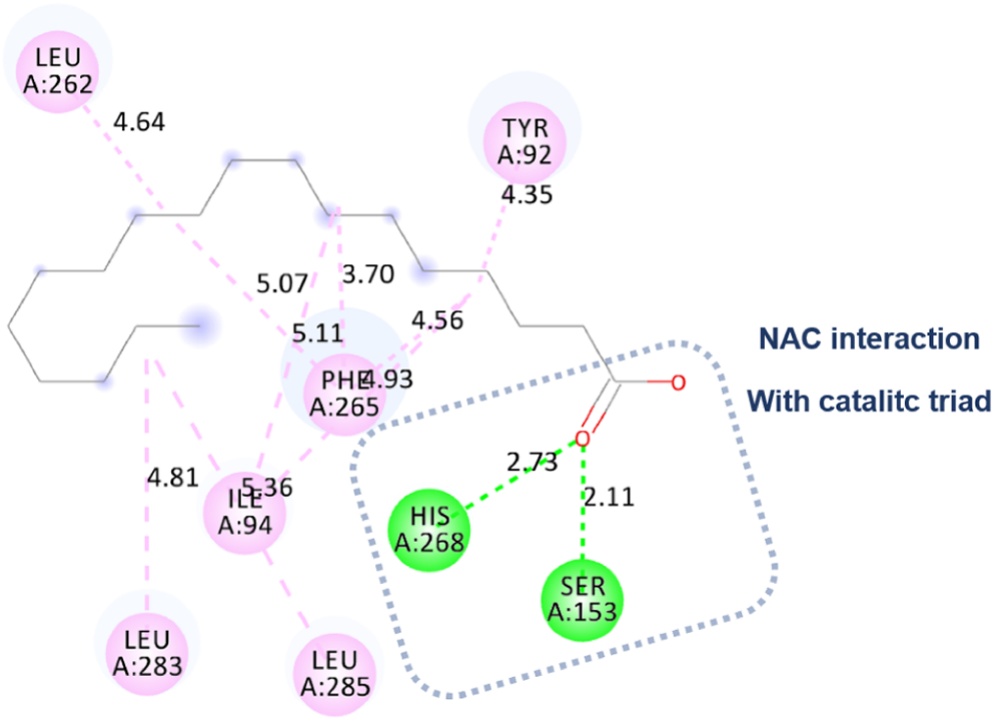
Molecular docking: approximation of octadecanoic acid and the Eversa 2.0 region, with an approximation of the catalytic site of the lipase with the area where hydrolysis would occur.

The molecular docking simulation of hexadecenoic acid showed that the ligand was positioned very close to the catalytic triad of Eversa 2.0. However, no interaction with the residues of this triad was found. Instead, a hydrogen bond was formed with Tyr 29 (2.44 Å) due to the proximity between the carbonyl group of the acid and this residue. Other significant hydrophobic interactions, which may have driven the reaction, were observed. These interactions included alkyl-type interactions with residues Tyr 92, Ile 94, Phe 265, Val 269, and Leu 283 (Figure 10).

**Figure 10:**
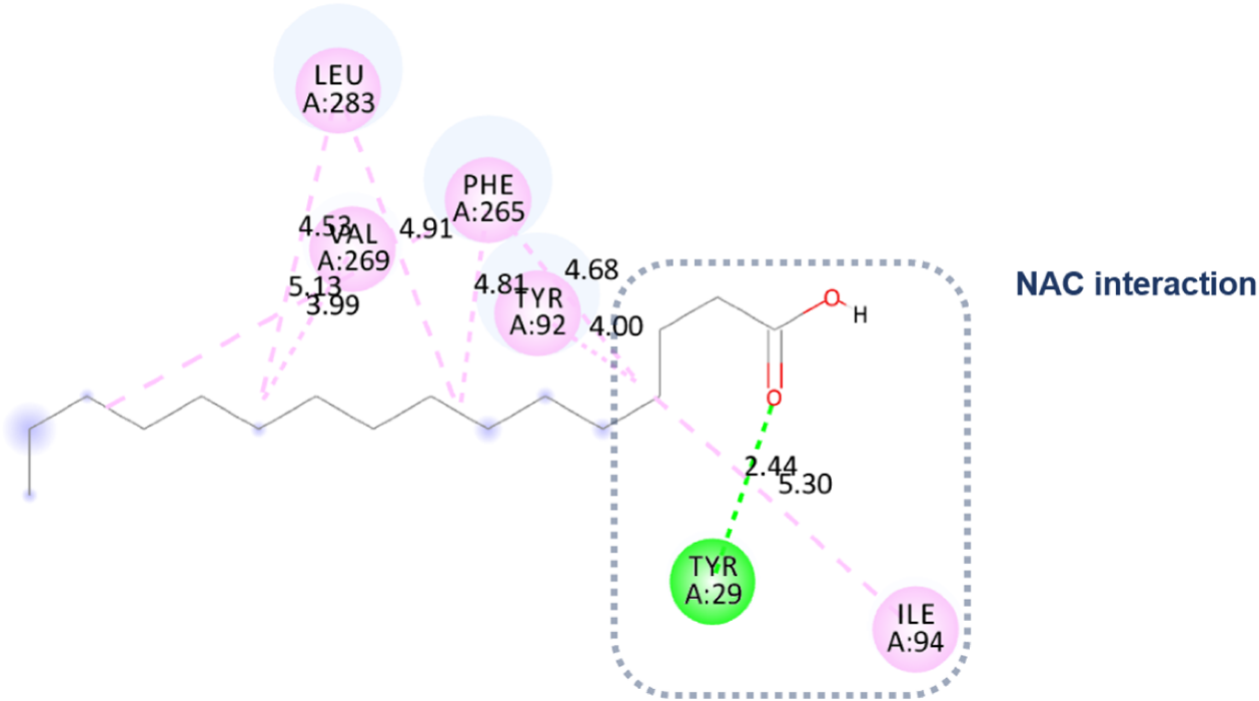
Molecular docking: approximation of hexadecenoic acid and the Eversa 2.0 region with an approximation of the Eversa 2.0 catalytic triad, with no record of residues from this triad.

Finally, conclude this study, the simulation for the mixture of CALB plus and Eversa 2.0 lipases demonstrated an approximation of the lipase catalytic site to the region where CALB hydrolysis would occur. Consequently, the 10 docking positions poses were classified as strong NACs because the distance between Ser-O (105) and the carbonyl-C of the substrate was less than below 3 Å (Figure 9), with an angle less than 60°, and an additional hydrogen bond with His 224 (Figure 9).

In the case of dodecanoic acid, which can also be observed in Figure 11, a hydrogen bond was observed with a residue outside of the catalytic triad, specifically Thr 40 (2.91 Å), along with Thr40 (2.91 Å), also an H-bond to the carbonyl group of the group’s oxygen (Figure 11). Val 154, Ile 285, and Ile 189, as well as hydrophobic interactions, were noted through alkyl-type residue interactions.

**Figure 11:**
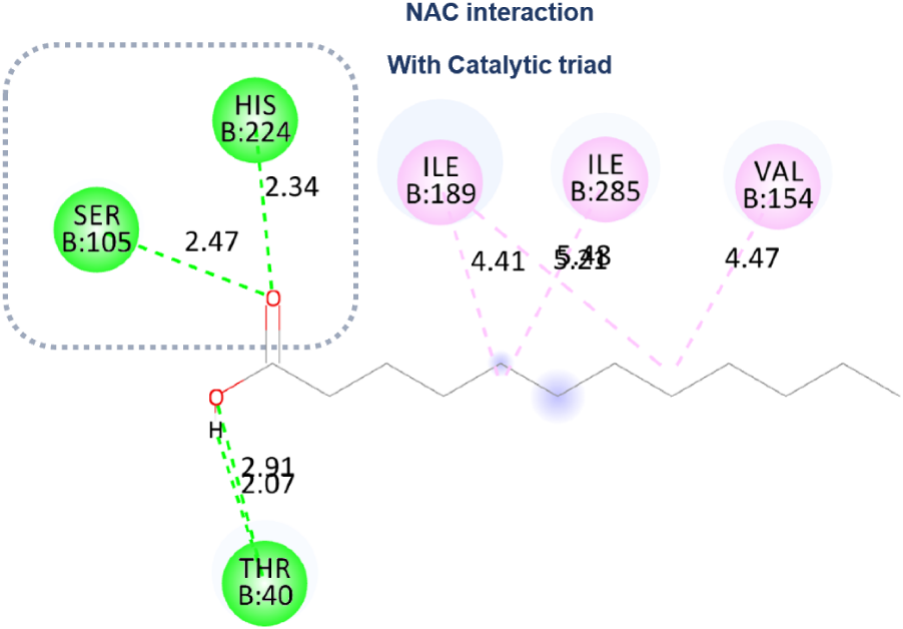
Molecular docking: Dodecanoic acid makes a hydrogen bond to a residue not part of the catalytic triad.

#### 3.6.2 Molecular Dynamics Simulations

##### 3.6.2.1 Root Mean Square Deviation (rmsd)

Ligands: The ligands studied were correctly accommodated in the catalytic site of l lipase CALB and Eversa.^114^ Simulation studies were performed on the lipase-ligand complexes (Figure 9) and were conducted to evaluate not only the conformational changes of the enzyme but also its stability after each conformational change. During the simulation, the Root Mean Square Deviation (RMSD) of the lipase–ligand complexes was used to assess the extent of conformational changes in the studied molecule. Figure 12 shows the RMSD behaviors of the studied complexes during the equilibration phase, stage.^114^

**Figure 12:**
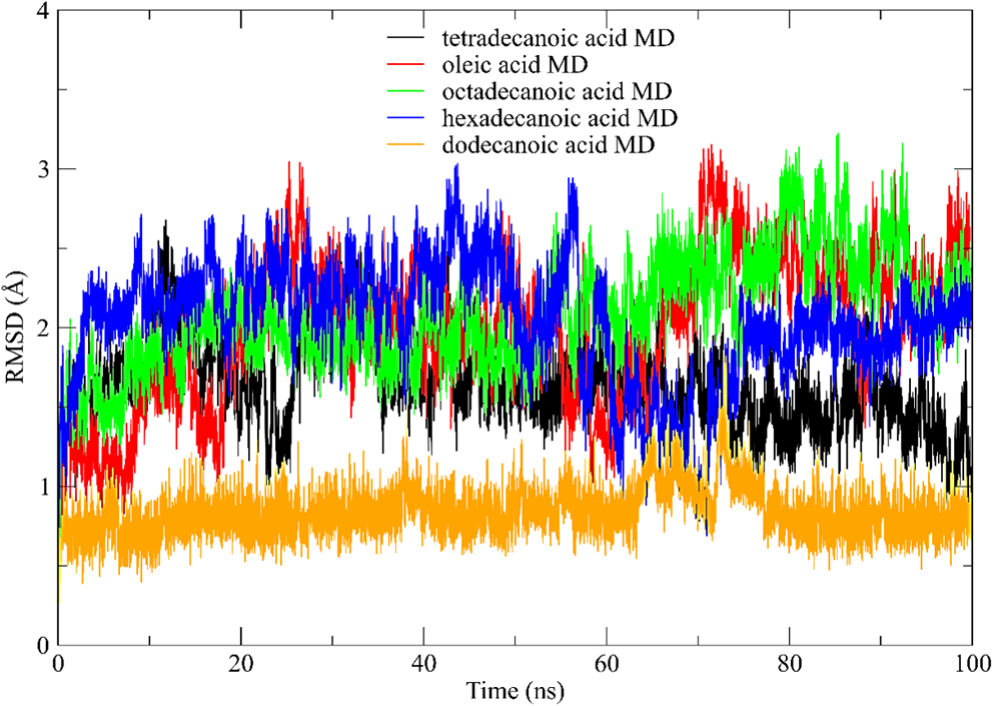
Root Mean Square Deviation (rmsd) for the initial conformation of the complexes versus the simulation time (nanoseconds) in the equilibration step. tetradecanoic acid (black); oleic acid (red); octadecanoic acid (green); hexadecenoic acid (blue) and dodecanoic acid (orange).

From the equilibrium simulations of the lipase–ligand complexes in the solvent, preliminary information on the behavior of the conformations during the dynamics was obtained. It was observed that the RMSD stabilization values of the studied ligands oscillated between 0.8 and 2.3 Å over the evaluated time. These low rmsd values may be associated with the movement of ions and solvents in the system during the initial conformation of the complexes. Figure 12 presents the results obtained during the production stage.

We obtained preliminary information on the behavior of the conformations for the dynamics from the equilibrium simulations of the lipase-ligand complexes in the solvent. At this step, rmsd values of the studied ligands oscillated between 0.8 and 2.3 Å, which can be related to the movement of ions and solvents in the system during the initial conformation of the complexes. Figure 12 shows the results obtained in the phase of production.

As shown in Figure 12, dodecanoic acid had average rmsd values below 1.0 Å, while tetradecanoic acid had presented values around 1.0 Å. The other ligands showed values between 2.0 and 2.3 Å. This work’s theoretical and experimental results indicate that fatty acids from residual oil form stable complexes with the catalytic site of Eversa and CALB complexes, suggesting a viable alternative for future applications.

DataIt is worth noting that the data presented in the present study are consistent with results obtained in the literature, where studies have evaluated the affinity and molecular stability of different fatty acids with lipases in esterification reactions to the present study. The authors observed that previous studies found that octadecanoic acid had one of the highest rmsd values and, hence, one of the lowest, indicating lower stability than other complexes.^114^

##### 3.6.2.2 Hydrogen Bonds

Intermolecular hydrogen bonds play a crucial role in maintaining the stability of protein–ligand complexes, making them a focal point of central focus in biochemical research. This investigation explored the importance and significance of these bonds in sustaining the stability of lipase–ligand complexes and was investigated using molecular dynamics simulations to unravel and reveal the dynamic nature of these interactions.

The analysis primarily focused on the scrutiny of the formation of the intermolecular hydrogen bonds between the ligands and lipase during both the equilibration and production phases. Figure 13 from the study illustrates fluctuations in the hydrogen bond networks throughout these stages, highlighting the variations in bond formation.

**Figure 13:**
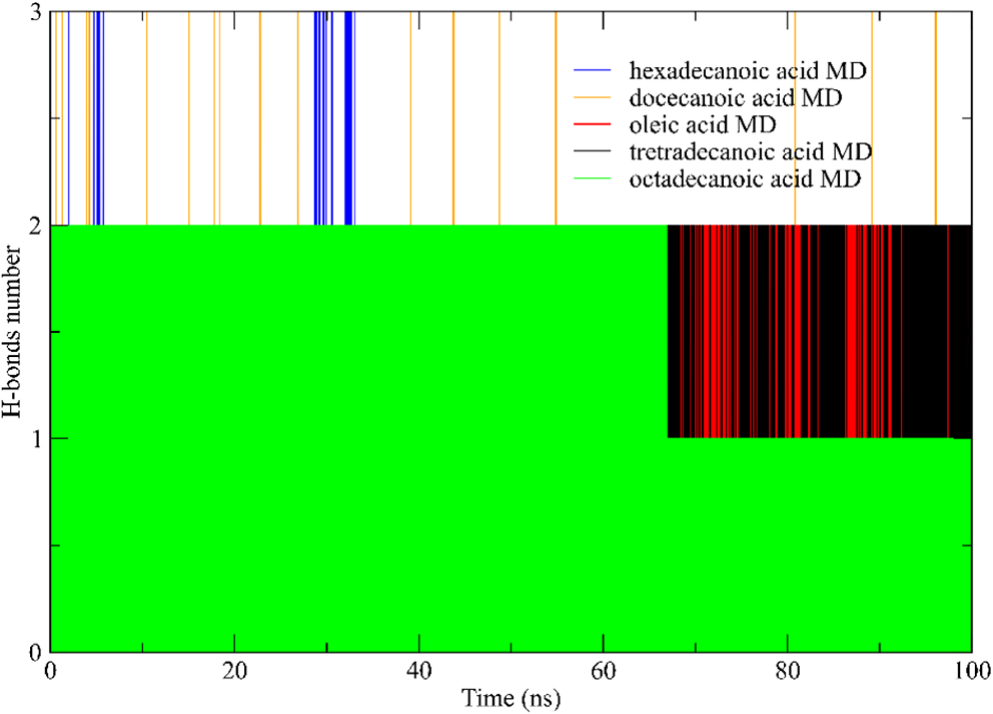
Hydrogen bonds formed between the protein and the ligand during the two simulation steps. tetradecanoic acid (black); oleic acid (red); octadecanoic acid (green); hexadecenoic acid (blue) and dodecanoic acid (orange).

A notable finding was the fluid nature of hydrogen bond formation, with the number of bonds oscillating between 1 and 3 during the simulation. Dodecanoic and hexadecenoic acids were particularly interesting, demonstrating up to 3 hydrogen bonds over the 100 ns trajectory.

The rupture of hydrogen bonds signaled indicated shifts in stability changes, often attributed to interactions such as van der Waals forces or hydrophobic forces. Additionally, the analysis revealed a direct correlation between the length of the ligands’ carbon chains of the ligands and the average number of hydrogen bonds, confirming results supporting insights from previous molecular coupling docking studies^114^

A key revelation from this investigation was the distinction between dynamic (molecular dynamics) and static (molecular docking) processes. While molecular docking provides valuable insights into static interactions, molecular dynamics simulations capture these complexes’ dynamic evolution, offering a more comprehensive understanding of their stability.

Moreover, the excess of additional exposed bonds observed in molecular dynamics simulations could be attributed to interactions with solvent molecules, underscoring the need to consider the importance of solvent effects when studying protein–ligand interactions.

This investigation provides valuable insights into the dynamic interplay of intermolecular hydrogen bonding bonds in lipase–ligand complexes. By revealing the dynamic aspect of these interactions, the study enhances our understanding of protein–ligand recognition processes, with implications for drug design and efforts in enzyme engineering endeavors.

## 4 Conclusion

Advancements in low-energy catalytic processes have the potential to significantly influence future developments in science, technology, and industry, particularly in terms of sustainability and energy consumption. Global collaboration is crucial for developing new methodologies and technologies that improve growth conditions, prevent contamination, and optimize oil extraction. The bibliometric mapping of existing literature plays an essential role in informing regulatory policies and guiding future research. By integrating these findings, the authors can conduct comprehensive assessments and strengthen their recommendations for large-scale biodiesel production using advanced catalysts.

Molecular docking analysis of the CALB plus Eversa 2.0 lipase mixture revealed a close indicated proximity between the catalytic site of the lipase and the region susceptible to where CALB hydrolysis. This observation highlights the occurrences, highlighting ten key NAC positions as crucial candidates for potential NACs. The resulting. The results of these complex findings were thoroughly evaluated through molecular dynamics simulations.

During molecular dynamics, the ligands studied adequately accommodated in the CALB and Eversa 2.0 lipase catalytic sites. It was observed that the simulations. The formed complexes demonstrated high stability throughout the preparation production and matching stages. The combilipases–combilipase–substrate complexes exhibited low RMSD values, indicating that the selected poses were suitable for the study. Additionally, both theoretical and experimental results suggest that fatty acids from waste frying oil form stable complexes with the catalytic sites of the CALB and Eversa 2.0 combi-lipases (Ser 153, His 268, and Asp 206), indicating a viable alternative for future applications.

The primary bottlenecks to the commercial viability of this process are related to scalability, cost-effectiveness, and the optimization of catalyst efficiency in large-scale operations. Furthermore, there are notable research gaps concerning the long-term stability and reusability of catalysts and integrating this process with existing biodiesel production systems.

Future research efforts must focus on addressing these challenges, including the further development of advanced catalysts, enhancement of the process’s energy efficiency, and the execution of more comprehensive life cycle assessments (LCA) to assess the environmental impact. Additionally, collaboration between researchers, industry stakeholders, and policymakers will be critical in identifying appropriate regulatory frameworks and technological innovations to facilitate the transition to large-scale commercial production.
